# Pain in Advanced Stages of Dementia: The Perspective of Medical Students

**DOI:** 10.3390/medicina55050116

**Published:** 2019-04-26

**Authors:** Agnieszka Neumann-Podczaska, Slawomir Tobis, Lyudmila Yermukhanova, Katarzyna Wieczorowska-Tobis

**Affiliations:** 1 Department of Geriatric Medicine and Gerontology, Poznan University of Medical Sciences, 60-781 Poznan, Poland; ar-n@wp.pl; 2 Department of Public Health and Health Care, West Kazakhstan Marat Ospanov State Medical University, Aktobe 030019, Kazakhstan; aleka_2807@mail.ru; 3 Department of Palliative Medicine, Poznan University of Medical Sciences, 61-245 Poznan, Poland; kwt@tobis.pl

**Keywords:** education, pain, dementia, medical students, knowledge

## Abstract

*Background and objective:* The number of studies related to medical students’ attitude toward pain is limited. The aim of our study was, thus, to assess the medical students’ knowledge of pain assessment and treatment in advanced stages of dementia in order to improve the existing curriculum in this area. *Material and methods*: We analyzed the medical students’ knowledge about pain in advanced dementia based on a short questionnaire. The research was anonymous. The questionnaire was completed by 147 students. *Results*: The students most often suggested that pain in patients with advanced dementia could be manifested via body language and facial expression (107 students—72.8% and 100 students—68.0%, respectively). Vocalization was the third most frequently reported pain manifestation (84–57.1%). Other groups of pain symptoms (changes in activity patterns, changes in interpersonal interactions, and mental status changes) were indicated less often (*p* < 0.0001). Only five students (3.4%) listed the DOLOPLUS behavioral pain scale as an assessment tool for patients with advanced dementia, and 16 (10.9%) indicated observational scale elements or a necessity to observe the patient. Still, 110 students (74.5%) correctly characterized pain treatment in patients with advanced dementia. *Conclusions*: To the best of our knowledge, our study is pioneering in defining the deficits of medical students’ knowledge on pain assessment and treatment in patients with advanced dementia. We highlighted knowledge gaps in the area of pain assessment which might make medical students incapable of proper pain treatment. Following the International Association for the Study of Pain considerations regarding the need for excellence in pain education, these results can contribute to the improvement of existing medical curricula in Poznan University of Medical Sciences to include pain management in dementia in a more ”patient-centered” way in order to increase future staff’s competency and to assure a better quality of care.

## 1. Introduction

It is estimated that dementia is accompanied by pain in as many as 18.0–83.8% of patients [1,2]. The discrepancy in numbers is at least partially related to difficulties in proper pain recognition. As patients with severe dementia are unable to communicate verbally and to consequently describe their pain, it is necessary to implement observational tools and behavioral indicators dedicated to these subjects. The use of these tools, however, requires a specific training of the healthcare providers [3,4,5].

While both the causes and incidence of pain in dementia are comparable to those without dementia diagnosis, the former are at greater risk for both an underassessment and undertreatment of pain. The American Geriatric Society reported a twofold decrease in the likelihood of opioid analgesia for each five-point decrease in the Mini-Mental State Examination score in patients undergoing a hip fracture surgery [6]. Monroe et al. also showed that nursing home residents without a dementia diagnosis were more likely to have an analgesic prescription for their pain, compared with the residents with a dementia diagnosis [7].

There are many negative consequences of inadequate pain management [8]. The exacerbation of existing cognitive impairments or behavioral disturbances are among them [9]. They may additionally worsen verbal communication skills, consequently leading to further pain misdiagnosing [10].

In patients with advanced dementia, both pain identification and its treatment rely on the ability of the staff to interpret the patient’s reactions [11]. As a deficient knowledge of healthcare professionals regarding pain in dementia has been observed [12,13], they ought to gain the necessary skills and knowledge to enhance their pain management practices. Moreover, the development of positive attitudes and perceptions among students is a major goal of preservice medical education. It is henceforth important to understand the educational needs related to pain in dementia.

Notably, the number of studies related to students’ knowledge and attitude toward pain is limited. To the best of our knowledge, there have no studies been published related to the students’ knowledge and attitude toward the pain in patients with advanced dementia. Hence, the aim of our study was to fill this gap to be able to improve the existing curricula in this area.

## 2. Materials and Methods

The study was approved by the Bioethical Committee of Poznan University of Medical Sciences in Poznan, Poland (KB no 1204/18).

Its participants were recruited among the 8th semester medical students of Poznan University of Medical Sciences at the beginning of an eight-hour class devoted to delirium. In Poland, the whole medical education lasts 12 semesters.

The students’ knowledge about pain in advanced dementia was analyzed based on a short questionnaire; they were asked to abstain from using any external sources, including the Internet, when answering the questions. Therefore, it is reasonable to assume that the answers provided reflected their knowledge. The research was anonymous.

We assumed that at least every second medical student of the 8th semester would be included in the study. All who were asked did participate. The questionnaire was completed by 147 students (55.1% of the total group). It consisted of 3 questions, formulated in an open manner, so as not to suggest answers. These questions were created by two independent experts in the fields of geriatric and palliative medicine (MD, Professor) and clinical pharmacology (PhD). Both of them are graduates and members of the European Academy for Medicine of Ageing (EAMA). The consensus between experts was achieved through discussion. The questions were as follows:

1. What are the clinical symptoms of pain in patients with advanced dementia?
2. Which scales would you use while assessing the pain in patients with advanced dementia?
3. Are the pain treatment rules in patients with advanced dementia similar to those without cognitive impairment? If yes, why? If no, why?

The answers to the first question were grouped according to the American Geriatric Society Panel on Persistent Pain in Older Persons [6] which outlined six domains that should be incorporated into the behavioral pain assessment chart:

- facial expression,
- negative vocalization,
- body language,
- changes in activity patterns,
- changes in interpersonal interactions, and
- mental status changes.

Answers to the second question after the initial analysis were grouped as follows:

- observation of the patient including observational pain scales—Abbey Pain Scale [14] and DOLOPLUS scale [15]
- pain assessment methods and pain scales recommended for use in the general population, including subjects with mild and moderate cognitive impairment (Visual Analogue Scales (VAS), Numerical Rating Scale (NRS), or Face Pain Scale (FPS) [16].

Based on the answers given to question three, the respondents were divided into three groups as follows:

- students who answered yes,
- students who answered no, and
- students who answered yes and no.

Next, all answers (along with their explanations) were analyzed in detail. The given explanations were grouped as follows:

- correct,
- partially correct, and
- wrong.

The analysis of the qualitative data was performed by a geriatrician—a graduate and member of the European Academy for Medicine of Ageing (EAMA)—and independently by a pharmacist (also an EAMA graduate and member). In the case of discrepancies, the answers were discussed to obtain a consensus.

### Statistical Analysis

The results were expressed as the percentage of provided answers or as mean ± standard deviation and median + range (due to a lack of normality). To evaluate the normality of distribution, the Shapiro–Wilk test was applied. A comparison between the groups was made by means of the ANOVA test with a post hoc Dunn test. The statistical significance was set at *p* < 0.05.

## 3. Results

The studied students most often suggested that pain in patients with advanced dementia could be manifested via body language and facial expression (107 students—72.8% and 100 students—68.0%, respectively). Vocalization was the third most frequently reported pain manifestation (84—57.1%). Other groups of pain symptoms were listed less often (changes in activity patterns: 38 students—25.8%, changes in interpersonal activity: 49 students—33.3%, and mental status changes: 69 students—46.9%).

On average, students declared symptoms from 3.0 ± 1.2 analyzed pain areas (median 3.0, range 0–6). Only five students (3.4% of the respondents) did not suggest any symptoms from any domains. However, one of those students claimed, “silent pain; pain response is the same as the child’s one—visible, understandable, natural and primary.” Only four respondents (2.7%) listed symptoms from all six areas.

As far as pain assessment tools for patients with advanced dementia are concerned, only five students (3.4%) mentioned the DOLOPLUS scale and as few as 16 (10.9%) indicated observational scale elements or the necessity to observe the patient (i.e., “I would apply the scale depending on the patient’s condition” and “I would conclude based on the clinical picture, e.g., tachycardia, sweats, screams in pain suggest that pain is exacerbated”).

As many as 93 students (59.9%) mentioned unilateral pain scales dedicated to the general population, including 53 respondents who indicated the FPS scale (as being valid alone or in combination with NRS or VAS) and five (3.4%) who listed both unilateral pain scales and patient’s observation. Another 35 students (38%) stated they did not know how to assess pain in patients with advanced dementia, including one who expressed lack of knowledge but explicitly ruled out using the VAS or NRS scale.

No association was observed between the number of domains in which students reported pain symptoms and the responses given for the pain assessment method in subjects with dementia (observation: 3.0 ± 1.1, median: 3.0, range 1–6; unilateral pain scales: 3.0 ± 1.3, median: 3.0, range 0–6; I do not know: 2.9 ± 1.0, median: 3.0, range 1–5).

Regarding pain management in patients with advanced dementia, only 30 students (20.4%) claimed that the rules of pain treatment were the same as in other patients. Additionally, 11 students (7.5%) gave a double answer—indicating that the rules of pain management in some aspects are the same, but in others, they differ.

Nevertheless, a detailed analysis of the explanations given by those students who believed that pain treatment rules are different for patients with advanced dementia showed that these answers indeed were correct. According to the studied subjects, differences resulted from, e.g., the need to observe the patient (by the staff), as they are not able to report their pain, or the necessity to administer drugs to patients due to noncompliance.

In total, it was found that only 16 students (10.9%) gave incorrect answers, six (4.1%) did not answer at all, and 15 (10.2%) gave partially incorrect answers (e.g., other perception of pain, no reporting of breakthrough pain, more frequent interactions, noncompliance).

Thus, 110 students (74.5%) correctly characterized the pain treatment in patients with advanced dementia. Examples of correct answers: *YES, the pain treatment rules are the same* and the answers *NO*, *the pain treatment rules are different*, with both correct and incorrect explanations, are presented in Table 1.

## 4. Discussion

The topic of analgesia in patients with dementia is given more and more attention, and the necessity to introduce pain evaluation and monitoring of analgesia appropriateness as a standard procedure in the geriatric assessment is widely emphasized [3,4,11,12,13,14,15,16,17]. The present study is pioneering in the verification of the medical students’ knowledge regarding pain in advanced dementia. We found that one in every four students would not treat pain in these subjects properly. Students, e.g., stressed the need to modify the dosing rules in the presence of advanced dementia (including both increasing and decreasing analgesics’ doses). They also expressed concern regarding the treatment-related risk in subjects with advanced dementia due to the age-related changes in the metabolism of drugs.

Zwakhalen et al. [13] observed a poor knowledge of medical students regarding analgesics’ dosage. A similar knowledge gap was also reported in the study of Barber and Gibson [18] who showed that physicians appeared to be uncertain about the optimum management of pain in dementia due to the beliefs on age-related changes in drug metabolism. Knowledge gaps in the area of pharmacological treatment may contribute to the undertreatment of pain.

Regarding pain detection, we showed that almost all medical students knew selected symptoms of pain in patients with advanced dementia. Among them, facial expression, negative vocalization, and body language were pointed out the most frequently. These symptoms seem to be consistent across the lifespan [19], and their assessment does not require the evidence of prior data or trends, which can make students more familiar with them. Conversely, such symptoms as changes in behavior and mental status require either the knowledge of prior behavioral patterns and a constant observation or seeking information from others (family members and further healthcare professionals). Thus, they require a more difficult assessment strategy, which possibly makes the students less likely to indicate them. It is in line with the observation of Zalmay and Williams [20] who determined how medical students used and understood pain rating scales. They showed that, although medical students’ recognition of the importance of facial expression was encouraging, they expected a narrow range of other pain-associated behaviors. Moreover, both facial expression, negative vocalization, and body movement should not be used alone as pain indicators, as patients can present them for other reasons, i.e., discomfort or fear [21].

It has been shown that behavioral disturbances resulting from pain in dementia are often misdiagnosed [22,23,24] and are primarily perceived as psychiatric or psychological issues. Hence, the first response to behavioral disturbances is often the administration of psychotropic drugs [22]. Their adverse reactions may mask symptoms of pain and, thus, create a barrier to efficient pain assessment [10]. One may conclude that proper pain assessment and management are essential to avoid the excessive use of sedatives, [9,25,26], making the education in these areas very important.

In our study, only one of every ten students pointed out an observational scale or its elements whereas the rest listed unilateral pain scales (VAS, NRS, and FPS). This may reflect the lack of official standards concerning the use of pain assessment tools across all settings dedicated to subjects with advanced dementia [3]. It is in line with the observation of Barry et al. [27] who showed that British Pain Society guidelines on pain diagnosing methods and treatment in the UK are underused or even not used at all.

On the other hand, it must be pointed out that Visual Analogue Scale, Numerical Rating Scale, and Face Pain Scale are claimed as valid for older people with moderate to severe cognitive/communication impairment as long as the subjects still understand them [28]. Moreover, while unilateral pain scales are frequently used and are very popular in everyday practice, observational scales are not. All this translates into a risk of pain under-assessment and, again, increases the importance of proper education.

Our study also has some limitations. We did not use a standardized tool to assess medical students’ knowledge and attitude toward pain in advanced dementia since—to the best of our knowledge—such a tool does not exist. Hence, no validation of our questionnaire against a golden standard tool was possible. Importantly, the scheme of our tool provides for not suggesting answers which is substantial for its objectivity. The other limitation is that our study was a cross-sectional analysis with no intervention. Still, we aimed to define the knowledge gaps before the course to know what are the most important topics which need to be covered.

As far as the pre-graduate education is concerned, knowledge deficits regarding pain in patients with advanced dementia highlight the need for a practical confrontation of medical students with patients. It could belong to the educational strategy to prevent the application of inadequate pain assessment tools as well as to ensure the proper use of behavioral tools. Also, there is a need to close the knowledge gap in the pharmacological treatment of pain and to reorganize curricula to include pain as a disease state not merely the symptom.

## 5. Conclusions

In conclusion, to the best of our knowledge, our study is pioneering in defining the deficits of medical students’ knowledge on pain assessment and treatment in patients with advanced dementia. We highlighted knowledge gaps in the area of pain assessment which might make medical students incapable of proper pain treatment. Following the International Association for the Study of Pain considerations regarding the need for excellence in pain education, these results can contribute to the improvement of existing medical curricula in Poznan University of Medical Sciences to include pain management in dementia in a more ”patient-centered” way in order to increase future staff’s competency and to assure a better quality of care.

## Figures and Tables

**Table 1:** Examples of a pain treatment’s characteristics.

Answer YES	Answer NO with Correct Argumentation	Answer NO with Incorrect Argumentation
“yes, they feel pain in the same way”	“no, because the symptoms may be atypical and it is more difficult to assess pain in patients with dementia”	“no, due to side effects, another metabolism”
“yes, dementia does not affect the action of drugs”	“no, because there are problems with communication and self-medication”	“no, more medicines that the patient takes, slowed metabolism [cause] interactions, other pharmacodynamics and pharmacokinetics; the underlying somatic causes are also different”
“yes, goals, medications and dosage [are] the same”	“no; the patient is unable to describe the pain, the emphasis [should be] on verifying the treatment of pain”	“no, due to the high burden of other conditions, which causes taking of a broad spectrum of drugs and drug interactions; they are more likely to have side effects; lower doses are often effective”
“yes, patients with dementia suffer the same as those without, and one should seek the same pain control”	”no, because the treatment is based on observation of the patient, a different manifestation of pain, lack of reporting of the need to increase the dose by the patient, difficult to assess the pain, patients [are] difficult to determine the doses”	“no, we do not give drugs orally”
“yes, dementia does not affect the choice of drugs, the most important is pain relief’	“no, the NRS scale is unreliable, [it is] more difficult to assess the effects of treatment and contact with the patient”	“no, lower doses are needed, more carefully introduced”
“yes, we use the analgesic ladder plus observation of the patient for doses, adverse effects and effects of treatment as well as chronic and breakthrough pain”	“[the patient] is unable to take the medication on their own, one cannot rely on the patient’s opinion on the effectiveness of the treatment, more difficult assessment of pain and of dosage”	“no, doses are higher in the group of patients with advanced dementia”
